# Skin Conductance Responses Indicate Children are Physiologically Aroused by Their Favourite Branded Food and Drink Products

**DOI:** 10.3390/ijerph16173014

**Published:** 2019-08-21

**Authors:** Rachel Smith, Bridget Kelly, Heather Yeatman, Stuart Johnstone, Louise Baur, Lesley King, Emma Boyland, Kathy Chapman, Clare Hughes, Adrian Bauman

**Affiliations:** 1 Early Start, School of Health and Society, Faculty of Social Sciences, University of Wollongong, Wollongong, NSW 2522, Australia; 2 School of Health and Society, Faculty of Social Sciences, University of Wollongong, Wollongong, NSW 2522, Australia; 3 Brain and Behavior Research Institute, School of Psychology, Faculty of Social Sciences, University of Wollongong, NSW 2522, Australia; 4 Discipline of Child and Adolescent Health, Sydney Medical School, University of Sydney, Sydney, NSW 2006, Australia; 5 Sydney School of Public Health, Sydney Medical School, University of Sydney, Sydney, NSW 2006, Australia; 6 Psychological Sciences, Institute of Psychology, Health and Society, University of Liverpool, Merseyside L69 7ZA, UK; 7 Cancer Council NSW, 153 Dowling Street, Woolloomooloo, NSW 2011, Australia; 8 School of Life and Environmental Sciences, Faculty of Science, University of Sydney, Sydney, NSW 2006, Australia; 9 School of Medicine and Public Health, University of Newcastle, Callaghan, NSW 2308, Australia; 10 Prevention Research Collaboration, School of Public Health, University of Sydney, Sydney, NSW 2006, Australia

**Keywords:** public health, food marketing, food packaging, electrodermal activity, childhood obesity, skin conductance responses, food and beverage branding

## Abstract

Children’s favourite food and beverage brands use various tactics to foster positive associations and loyalty. This brand-consumer dynamic is frequently influenced by the use of implicit techniques and emotional appeals. Few studies have used physiological methods to examine the connections that brands build with children and the influence this has on their automatic responses. These techniques are potentially less prone to bias than behavioural or cognitive methods. This is the first study to explore the implicit response that children have to images of their favourite food and beverage brands using skin conductance responses as a marker of arousal. Australian children aged 8–11 years (*n* = 48) were recruited. Images of the participants’ favourite branded food and beverage products, alongside images of the same products unpackaged, their family and friends, and neutral objects were presented in a randomised order with a standard timed interval between images. Children were significantly more aroused by branded images of their favourite food and beverage products than by their unpackaged counterparts (*p* < 0.042, *d* = 0.4). The physiological response to the branded products was similar to the response to the children’s family and friends (*p* = 0.900, *d* = −0.02). These findings suggest that children may have an implicit connection to their favourite branded products.

## 1. Introduction

In Australia, one-quarter of children aged 5–12 years are overweight or obese [1]. Nutrition in childhood shapes lifelong eating habits and subsequently long-term health, as youth obesity is likely to persist into adulthood [2]. Changes within the environment are one of a number of significant factors contributing to recent increases in overweight and obesity [3], through surroundings and opportunities that promote obesity, known as the ‘obesogenic environment’ [4]. A salient characteristic of modern society is the ubiquity of marketing, with marketing campaigns being so common that they are normalised into everyday experiences [5]. The marketing of unhealthy food and beverages is an example of an environmental prime, whereby food-cues encourage over-consumption [6]. It is, therefore, a key driver of the obesogenic environment and restricting children’s exposure to unhealthy food marketing has been recognised as a global public health priority [7]. 

Children are an attractive target population for marketers for a host of reasons linked to the early establishment of brand relationships. For example, the development of brand loyalty, essential for maintaining consistent sales, is most lucrative when it begins at a young age, after which companies can anticipate a lifetime of loyalty and associated purchases [8]. As a result, brands aim to create long-lasting relationships with children by fulfilling their symbolic and hedonic needs [9]. This relationship is increasingly developed on an emotional level [10], with a number of studies noting the use of emotional appeals to engage children [11] and the staging of brands as tools for children to express themselves [12]. It is believed that from the age of two years old, children can identify and evaluate brands and products of preference when asking for presents [13]. Sequentially, as they grow, children begin to use brands to express themselves as they develop self-concepts and participate in self-appraisals of how they view themselves [14,15]. It is within the period of 7–13 years of age that emotional connections with brands increase in number and depth [15] and underlie long-term brand relationships [16].

Emotional advertising has also been highlighted as one of the most successful strategies in terms of sales and profit performance [17]. The use of implicit emotional appeal is problematic in defending against marketing’s persuasive appeal, as when marketing operates at a subconscious level, children cannot rely on ‘cognitive defences’ that may protect them from marketing. Cognitive defences are activated when children recognise and acknowledge the persuasive intent of marketing stimuli and through understanding the effects of exposure, are then motivated to resist it [18]. For example, the Food Defense Model requires that children actively recognise the marketing content being presented in order to initiate their cognitive defences [18]. This model thus contends that children would be unable to defend themselves if the marketing techniques subverted conscious processing. 

It has been well documented that food cues stimulate a set of automatic responses, including increased heart rate, gastric activity, saliva, and skin conductance level [19,20,21]. However, less is known about the automatic reaction children have to branded and packaged foods to which they are favourable. Exploratory research with food products [22] and food brand choice [23] using methods originally designed to measure explicit cognitive processing (questionnaires and overt behavioural measures), has indicated that there are potential personal emotions and preferences for branded foods. Marketers typically strategise to induce brand preference by conditioning and appealing to subconscious motives [24]. Given that at least some of children’s brand associations and attitudes are non-conscious, and this state is very difficult to retrieve consciously [25], subjective measures of emotional state can be problematic. Moreover, when subjective techniques are used to measure the impact of marketing, such as self-report, they are prone to social desirability bias, demand characteristics and peer influence. Methods that bypass conscious thought processes are less susceptible to these factors and therefore could be implemented to obtain objective data on children’s responses to branded food and beverages. 

As emotional appeals are documented as a frequently used tactic to engage children with food brands [11], it could be speculated that exposure to a liked brand or food product would be accompanied by an automatic emotional response. A ‘non-conscious’ measure of emotional response to food brands may be attainable through physiological and psychological changes such as autonomic and endocrine responses [25,26], whereby arousal is thought to partially originate from emotional processes [27]. Electrodermal activity (EDA) refers to the variation of the electrical properties of skin in response to sweat secretion and is used as a physiological arousal measure [28]. It includes both tonic (skin conductance level, or SCL) and phasic (skin conductance response, or SCR) measurements of electrical conductivity in the skin [28]. The eccrine sweat glands are involved in emotion-evoked sweating [29] and increased sweat gland activity has been documented as the body preparing itself for an appetitive response produced by a pleasant stimulus [30]. Measurement of psychological processes and the distinct neural mechanisms of pleasure thus may indicate the ‘liking’ of something [31]. 

The measurement of skin conductance has previously been used in the marketing field to quantify emotion-related aspects related to liked and disliked brands [32]. It has also been used in the health field to explore conditioned appetitive responses in adults [33,34] and to explore physiological responses to food packaging, whereby the findings concluded that the data could be used to anticipate product choice [35]. There are very few instances in the literature of the use of skin conductance with young children to explore their physiological responses to food [36] and the studies have not yielded conclusive findings to date.

A major benefit of measuring EDA is that it is simple and non-invasive, making it seem less intimidating than other physiological measures and a very suitable measure for use with children. Due to these factors, and the universal acceptance of EDA as an effective measure of emotional arousal [37], EDA presented as an appropriate measure for an implicit response to a branded food cue. 

The purpose of the current study was to implement the measurement of EDA on an exploratory basis to determine whether it could detect an implicit response to branded food products. To measure and explore the nature of a potential emotional response to branded food products, the study required a marker of an existing emotional response. Therefore, images of the participant’s friends and family were included in the study as an assumed existing emotional response to act as a guide to compare to in determining if there was an emotional response to the branded products. The same products, unpackaged, which are considered more directly appetitive and ‘ready-to-eat’ than foods in their packaging [38], were also included to compare to. If the findings showed a stronger response for the branded products, then this could indicate a learned response facilitated by food marketing. 

For this exploratory study, two novel hypotheses were developed to investigate the nature of the automatic response that children have to their self-nominated favourite brands. The favourite brands were packaged, therefore, they included several visual factors (e.g., colours) and other unknown factors (e.g., an image that may have sparked a memory) that had potential to contribute to an arousal response. However, this study aimed to explore branded packaging as a whole, inclusive of the various prompts, marketing techniques and context that ultimately led to the brand being selected. 

Specifically, it was hypothesised that

(1) SCRs to images of branded products would be larger than SCRs to images of unbranded (unpackaged with no brand identifiers) counterparts.
(2) SCRs to images of branded products would be similar to SCRs to images of the children’s family and friends.

## 2. Materials and Methods

Ethical approval for this study was obtained from the University of Wollongong Human Research Ethics Committee (HE16-233). Informed written consent from parents and guardians was provided, as well as verbal assent by the children before participation. Data were collected between September 2016 and July 2017. 

### 2.1. Participants

A total of 48 participants (31 male), aged 8–11 years (9.2 years ± 1.07) (mean ± SD) were recruited through opportunity and snowball sampling in the Illawarra region of New South Wales, Australia. This age group was selected because they were considered to be vulnerable to the influence of food marketing [39] and rely on superficial cues from brands to guide their product evaluations [40]. The parents of the child participants were initially engaged via flyers around the university campus, in school newsletters, and advertisements of the study were placed on social media using the Facebook Advert Manager. Following completion of the experiment, the parents of the participants were asked if they would recommend the study to other parents in their social networks. Socio-economic status was documented by recording participants’ postcodes. The SEIFA (Socio-Economic Indexes for Areas) Index of Disadvantage for Wollongong City and Shellharbour City [41] indicated that participants were from low (23%) and medium (77%) socioeconomic areas. This was slightly less disadvantaged than the average population in the recruitment locale [41].

### 2.2. Materials

The participants were given one week to supply five photographs of their favourite branded food and beverage products. Requesting images from participants has been employed previously in academic research in the form of a visual sociology technique called ‘Autodriving’. Autodriving is a photo-interviewing technique where in place of being given stimuli, the images are chosen by the participants themselves, indicating that the study is ‘driven’ by the photographer [42]. This was important to ensure that the images used were definitely relevant to the participants. 

A photo guide was provided and instructed that the images should be taken in the following way: the product had to be in its original packaging; there was only one product per photograph; there was a neutral background. Aside from the criteria listed in the guide, participants were free to choose what their favourite brands were to ensure that their choices were genuine and representative. The participants were also asked to provide five photographs of their closest family members and friends. A file for each participant was created consisting of five images for each of the following categories: Branded Food & Beverage, Family & Friends, Unpackaged Food & Beverage, and Filler images. The filler images were neutral images (not related to food or beverages), such as a brick wall, which were all sourced from the Open Affective Standardized Image Set [43] and had been categorized with low arousal ratings. The ‘Unpackaged Food & Beverage’ file referred to images of the participant’s favourite branded food and beverages without the packaging on (e.g., an unwrapped chocolate bar), which had been sourced from Google Images by the lead researcher (RS). All of the unpackaged stimuli were clearly recognisable, with a white background, and had no further branding on the product itself. 

### 2.3. Procedure

At the beginning of the visit, the participants were encouraged to feel comfortable with the equipment and once they understood the testing procedure, a baseline measure of EDA was recorded for two minutes (participants focused on a black cross travelling on the screen). Following this, the participants were asked to focus on the laptop screen for approximately five minutes whilst the images were randomly presented, and their EDA was measured. Upon completion of the study, the participants were thanked and given a $10 voucher as reimbursement for their time. 

### 2.4. The Paradigm for Arousal Measurement

A commercially available research-grade device (Simple Scope 2000, UFI, CA) measured the participant’s EDA for the duration of the experiment. Visual stimuli (20 images per participant) were presented on a laptop screen using the Presentation software package (Version 18.3, Neurobehavioral Systems, Berkeley, CA, USA). The 20 images were presented in a randomised order for a duration of 5 s, preceded and followed by a black screen for 8 s. Several factors influenced the duration time chosen for the stimuli display duration and black screen duration: the ‘unremarkable’ nature of the stimuli, the time needed for the SCR to occur and return to baseline levels, and the participant’s attentive state during the study (i.e., ensuring the participant did not become bored). As EDA is known to decline throughout the duration of a task [44], EDA was also recorded during the black screen (between each stimulus). 

Two Ag-AgCl electrodes (1080FG, UFI, Morro Bay, CA, USA) were taped to the distal phalanges of digits I and II on the participants’ non-dominant hands. A small amount of biopotential contact medium gel (Biogel 1090, UFI, Morro Bay, CA, USA) was applied to the electrodes and a constant voltage of 0.5 V was applied across the electrode pair. The experiment was conducted in a suitable environment for the EDA measurement: a quiet room that was free of distractions, with medium-level lighting, and at a temperature of approximately 22–24 degrees Celsius [28]. The participants were asked to sit still and limit movement of their hand to avoid disturbance in the EDA measurement. 

The Presentation software delivered stimulus markers to the Simple Scope device to record the precise moment that each event (i.e., image, blank screen) occurred, allowing accurate pairing of the events and physiological data. 

### 2.5. Data Analyses

EDA was recorded in microsiemens (μS). Data collected for each participant included the EDA during the pre-task baseline, stimulus delivery and black screens. The pre-task baseline data was not included in the analysis reported because the pre-stimulus baseline (black screens) provided the most appropriate and reliable measure of the child’s unstimulated arousal level, accounting for fluctuations throughout the task. The pre-stimulus baseline was also more accurate because the children had adjusted to the apparatus, whereas the majority of the pre-task baselines were unrealistically high due to heightened arousal and nerves from beginning the study. SCRs were quantified using a purpose-built script in MATLAB programming software [45]. The onset of a response to stimuli was categorised by a minimum 0.1 μS increase above the mean level of the last 4 s of the preceding pre-stimulus baseline. An SCR was considered valid if the following criteria were met: if the SCR showed an onset in the 1–4 s post-stimulus period [46] and if the SCR showed a peak during the 5 s subsequent to the onset [46]. SCR amplitude (SCR-amp) was calculated as the change from the onset of the response to the peak of the response [47]. SCR-amp was then averaged for each stimulus type for each participant and examined using IBM SPSS Statistics Package Version 25.0 for Windows [48]. As a Shapiro–Wilk test indicated that the data for each stimuli type was normally distributed, parametric statistical testing was used. 

A repeated measures analysis of variance (RM ANOVA) with a within-subject factor of stimulus type (Branded Food & Beverages, Family & Friends, Unpackaged Food & Beverages) was conducted on the SCR data. To test the hypotheses, data was analysed using two a-priori planned contrasts. The first contrasts compared the Branded Food & Beverages to Unpackaged Food & Beverages. The second contrasts compared Branded Food & Beverages to Family & Friends. As the contrasts were planned and there were no more of them than the degrees of freedom for the effect, no Bonferroni-type adjustment to α was required [49]. 

## 3. Results

**Figure 1:**
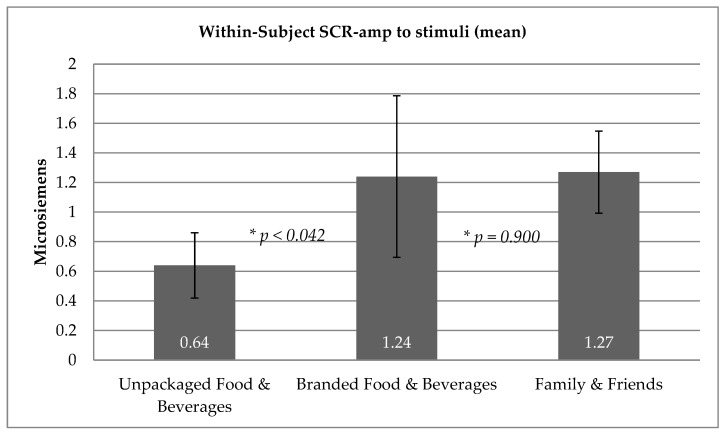
Skin conductance responses. * Indicates significance value between conditions.

The mean skin conductance amplitude (SCR-amp) for each stimuli type are detailed below in Table 1. The RM ANOVA with planned contrasts examined differences between stimulus types (*F*(3, 4.61) = 3.42, *p* < 0.19) (illustrated in Figure 1). The first contrast indicated that the SCR to images of Branded Food & Beverages (1.24 μS) was significantly larger than to the images of Unpackaged Food & Beverages (0.64 μS) (F (1, 47) = 4.37, *p* < 0.042). Cohen’s effect size value (*d* = 0.4) suggested a low to moderate practical significance. The second contrast indicated that there was no difference between the SCR to images of Branded Food & Beverages (1.24 μS) and images of Family & Friends (1.27 μS) (F (1, 47) = 0.16, *p* = 0.900). Furthermore, Cohen’s effect size value (*d* = −0.02) suggested a very low practical significance.

Skin conductance responses to Unpackaged Food & Beverages, Branded Food & Beverages and Family & Friends, with 95% confidence intervals.

## 4. Discussion 

This exploratory study sought to explore a potential implicit response emotional relationship that children may have with their favourite food and beverage brands, indicated implicitly by a physiological response to visual stimuli. The measurement of EDA highlighted contrasting responses to the same food and beverage products, depending on how they were presented, revealing a potential emotional connection that may underlie children’s favourite branded products. 

The most interesting finding was that there was evidence of a significant difference in children’s arousal when viewing branded food and beverage products compared to their unpackaged counterparts. The responsiveness to products in their branded packaging versus the actual product unpackaged was identified despite evidence that unpackaged foods can be perceived as more accessible, directly appetitive and have previously shown better physiological appetitive responses than packaged products [38]. This novel discovery supports the notion that children may have an emotional relationship with their favourite brands, which is a result of branding and marketing and is less determined by the product itself. 

Furthermore, similar arousal responses were found for images of the participant’s favourite branded products and images of their family and friends. The combination of these findings may help us to understand the strength of the connection that the participants have with their favourite food and beverage brands. These findings suggest that children felt a similar level of connection with their favourite food and drink brands to their family and friends. In other words, children may have exhibited the equivalent response to that of their relationship with their family and friends via a parasocial relationship that had developed with their favourite brands.

This finding is unsurprising as it has been previously noted that brands strive to make deep connections with children, particularly in the long-term, to control their loyalty to the brand over their lifetime [8,16,50]. As a result, it is likely that this response is due to the pervasive and powerful marketing techniques that are used to promote products to children, such as attractive packaging, which has been recognised as the strongest predictor of choice of packaged food products [51].

### 4.1. Strengths of the Study

Utilizing EDA, an implicit measure with child participants ensured that unbiased results that were free of social desirability could be obtained—a common drawback of using subjective methodology. 

Fifty percent of the images used in the study were directly selected by participants (specifically all of the favourite brand photos and family and/or friend photos), in contrast to the majority of previous studies that explored the influence of food marketing using pre-determined (researcher selected) food and beverage brand stimuli. The application of participant-nominated stimuli in this study assured that valid arousal stimuli were used in the evaluation of the associations and relationships that children may have with their favourite brands. 

### 4.2. Limitations

An imperfection of research that employs physiological methodology is the variation across individuals. Some individuals might respond with a larger increase in arousal with the EDA than other physiological measures, whilst other individuals might show smaller increases in arousal but greater changes in other measures [52]. Similarly, the nature of the current study may have been particularly stimulating or dull for different individuals. For some, it may have been un-arousing to sit and watch a somewhat repetitive screen for five minutes, thus their overall arousal level may have been low and the image categories may not have been stimulating enough to elicit a notable arousal change. Other participants may have experienced heightened arousal levels from taking part in the experiment and therefore, insignificant fluctuations in arousal may have occurred. 

In addition, the stimuli acquisition arrangement could have influenced the arousal responses. The participants were already familiar with the branded food images and family and/or friends photographs due to being involved with the capturing and reviewing of the images. Therefore, those photographs would have been familiar compared to the unpackaged photographs and children may have associated them with a positive experience. However, this did not necessarily determine the results, as familiarity could have also led to lower arousal due to a lack of novelty.

Furthermore, whilst they were not involved in the hypothesis testing, the filler images sourced from the Open Affective Standardized Image Set [43] and used to break the experiment up, initiated joint-highest arousal from the children (the same mean SCR-amp as Family & Friends). This was an unexpected finding as they were selected for their neutrality, however there could be a few potential explanations for this result. Since these images were not provided by the children, it could have been the unfamiliarity which led to high arousal—although this is not entirely clear because the other unfamiliar images (non-packaged foods) had the opposite effect, garnering the lowest arousal of all conditions. Alternatively, the high arousal may have been because the filler images were purposely unrelated to the rest of the stimuli, so this may have created more of an interest as they stood out more. Whilst this was not clearly determined, it is an important finding to note to the scientific community and should be considered by those who approach similar studies in the future. For example, in future studies, the filler image (if needed) could still broadly relate to the topic. 

Lastly, EDA only indicated the intensity of emotional arousal, so this study may have lacked an additional measure to capture emotional valence (positive or negative emotion), such as self-report, to potentially provide further insight into which stimuli were liked the most. However, this was considered unnecessary as it was assumed that participants would have a positive valence to images of their friends and family, favourite brands, and therefore, also to the images of the favourite brands unpackaged.

### 4.3. Implications for Future Research

The nature of children’s automatic responses to their favourite branded products, when compared to other stimuli such as their family and friends and unpackaged food and beverages, suggests that children have a strong implicit response to their favourite brands. Questions remain regarding further detail of these relationships. A natural progression of this work would be to consider further use of EDA in the exploration of emotional connections between children and food and beverage brands. For example, to fully understand the emotional relationship, it would be important to ascertain the age that the physiological response first manifests by measuring EDA in younger participants. 

It would also be interesting to understand what aspects of the branded products can induce a physiological response of liking. That is, there may be specific aspects of the brand imagery that are emotionally arousing or induce an appetitive response. It may be the case that specific persuasive appeals or food cues or cheeky content of food marketing may be responsible for triggering responses. EDA could next be used to investigate which particular marketing techniques produce a higher degree of response amongst common themes, such as the presence of cartoon characters, fun and humour. 

In recognition of the value of using physiological methodology in this area, other physiological measures could also be considered for their effectiveness at measuring implicit preferences for food and beverage brands. Alternative neurological methods, such as Functional Magnetic Resonance Imaging (fMRI) and the Electroencephalogram (EEG), could be considered. fMRI studies have been used for examining neural responses to product brands in children of a similar age [53] and EEG studies have shown that bipartite activations can indicate responses to positive and negative stimuli [54]. The frontal lobe asymmetry model allows consideration of EEG asymmetry and provides an index of approach motivation corresponding to greater activation on the left side of the brain. However, the participant burden needs consideration as the application of these methods is more complicated than for EDA and children would be required to remain extremely still for the apparatus to produce clear images. 

## 5. Conclusions

The implementation of a physiological measure illuminated another dimension to the response that children have with branded food and beverages. This study demonstrated that branded products generate an implicit response, greater than that of an unembellished food cue, a usually more appetising and biologically motivating food cue [38]. This response suggests that powerful marketing techniques have guided and fostered strong connections with children.

There is merit in using innovative methods in brand-consumer research, as methodology that achieves unfiltered and unbiased responses could reveal rich insights in this area. Given the feasibility and acceptability of the methodology, future research into brand associations and relationships should continue to investigate the implicit mechanisms that drive brand preferences and could consider implementing a range of physiological methods that will reveal other ways in which individuals respond to food marketing. 

## Figures and Tables

**Table 1:** Skin conductance responses for all stimuli.

Within-Subject SCR-Amp to Stimuli (Mean Microsiemens μS)			
Unbranded Foods and Beverages	Branded Food and Beverages	Family & Friends	Filler
0.64	1.24	1.27	1.27
